# The effect of first- and third-generation prophylactic antibiotics on hospitalization and medical expenditures for cardiac surgery

**DOI:** 10.1186/s13019-022-01763-4

**Published:** 2022-02-05

**Authors:** Sung-Jin Bae, Inah Kim, Jaechul Song, Euy-Suk Chung

**Affiliations:** 1 Department of Health Science, Hanyang University Graduate School, Seoul, Korea; 2 Department of Cardiovascular Surgery, Sanggye Paik Hospital, Inje University College of Medicine, Seoul, Korea; 3 Department of Occupational and Environment Medicine, Hanyang University College of Medicine, Seoul, Korea

**Keywords:** Prophylactic antibiotics, Surgical site infection, Propensity score matching, Medical expenditures

## Abstract

**Background:**

This study investigated the efficacy of first-generation (cefazolin) and third-generation (ceftizoxime) prophylactic antibiotics in patients undergoing cardiac surgery and the incidence of surgical site infections, hospitalizations, and medical costs.

**Methods:**

All adult patients (≥ 20 years) undergoing cardiac surgery at one hospital from January 01, 2009, to December 31, 2016, were included in this study. A single prophylactic antibiotic was administered at a dose of 1 g within one hour of the surgical incision and for three days after surgery at eight-hour intervals. After propensity score matching, 194 patients in each antibiotic prophylaxis group (first-generation vs third-generation) were analyzed. Among the 388 patients, the incidence of surgical site infections was compared according to the type of prophylactic antibiotic, and risk factors were evaluated by chi-squared tests followed by multivariate logistic regression analysis.

**Results:**

The incidence of deep surgical site infections was significantly lower in the first-generation group (5.7%) than in the third-generation group (16.5%). The pathogens isolated from the surgical infection sites were similarly distributed in both groups. However, the prevalence of highly infectious gram-positive bacteria was more than that of gram-negative bacteria (67% vs 23%). The preoperative hospitalization duration, mean operation time, and ventilator use time were similar in both groups, but the postoperative hospitalization duration was significantly shorter in the first-generation group (25.5 days) than in the third-generation group (29.8 days). In addition, the medical costs were lower in the first-generation group (20,594 USD) than in the third-generation group (26,488 USD).

**Conclusion:**

In conclusion, the first-generation prophylactic antibiotic was better than the third-generation in reducing surgical site infection rates, hospitalization length, and medical expenditures.

**Supplementary Information:**

The online version contains supplementary material available at 10.1186/s13019-022-01763-4.

## Background

In a placebo-controlled trial, the placebo group showed an increased incidence of surgical site infections by 20–50%, confirming the appropriateness of using prophylactic antibiotics in cardiac surgery [1–3]. Surgical site infections (SSIs) are common hospital infections that increase the morbidity and mortality of patients, treatment duration, and socioeconomic costs. According to the National Centers for Disease Control and Prevention (CDC) and the National Nosocomial Infections Surveillance (NNIS) report, surgical site infections account for 14–16% of hospital-associated infections in hospitalized patients [4]. SSIs increase the average number of days of hospitalization by 6.5 days, with additional hospitalization costs of $18,900, while the costs associated with patient mortality is $60,547 more than patient survival [5]. Korean studies showed that the length of hospitalization due to SSIs increased by 5.2 days, with an additional cost of more than $1800 per incident [6]. Therefore, SSIs lead to mental, physical, and economic losses to patients, worsens the quality of life, wastes healthcare resources, and increases the financial burden on medical institutions.

The results of the 2006 Survey on Antibiotic Usage from the National Health Insurance Review and Assessment Service (HIRA) showed that the use of prophylactic antibiotics in Korean surgeries differed from the Guideline for Guidance and has been classified as an abuse of antibiotics [7, 8]. The choice of prophylactic antibiotics is less appropriate for methicillin-resistant *Staphylococcus* sp. and can be covered by gram-positive bacteria and provides safe and cost-effective treatment but fails to reflect the domestic medical environment because it refers to guidelines from foreign clinical studies. In recent studies, sufficient medical institutions and research subjects were not available, which limited the generalizability of research results [9, 10]. Since the start of the national hospital evaluation program (NHEP) in 2008, the evaluation of prophylactic antibiotics for surgery was implemented as a comprehensive measure of antibiotic resistance management. In this evaluation program, unfavorable antibiotic choices were defined as the overuse of third-generation cephalosporin, aminoglycoside, combination of β-lactam with aminoglycoside, combination of vancomycin and other antibiotics. Procedures performed since 2008 were included in the NHEP assessment and the clinical performance results were officially reported to the public as well as to each hospital.

Third-generation prophylactic antibiotic, which was used from 2009 to 2012, was changed to first-generation prophylactic antibiotics due to the evaluation of prophylactic antibiotic use. The use of prophylactic antibiotics has been evaluated since 2012 to promote the prevention of SSIs. The benefit of university hospital institution in changing prophylactic antibiotics has not been identified. Therefore, we performed a comparative study of cephalosporin first-generation (cefazolin) and third-generation (ceftizoxime) antibiotics. The purpose of this study was to investigate the use of prophylactic antibiotics and the prevention of SSIs by analyzing the relationships between the use of prophylactic antibiotics and SSI rates.

## Materials and methods

### Study population

**Fig. 1:**
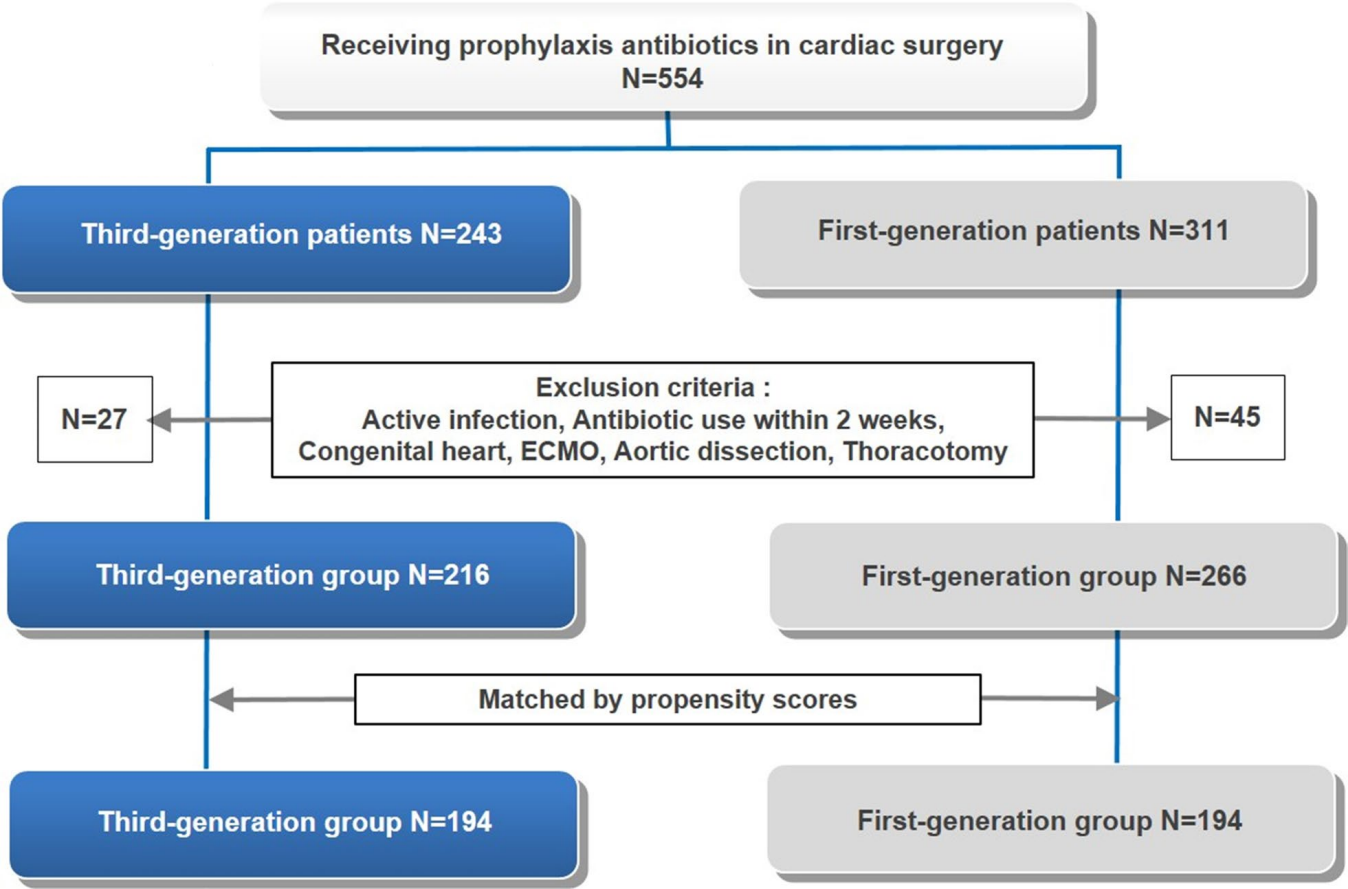
Selection of study subjects and the propensity score matching process. We reviewed the medical records of 554 individuals. After propensity score matching, 388 patients remained in the final analysis

This study was a retrospective review of the electronic medical records of all patients who underwent cardiac surgery from January 01, 2009, to December 31, 2016, at a single university hospital. All patients had undergone full median sternotomy and cardiopulmonary bypass (CPB). The inclusion criteria were coronary artery bypass grafting (CABG) and valve surgery. Patients with current active infections; those for whom antibiotics had been administered within two weeks of surgery; immunotherapy patients; patients with congenital heart disease, cardiac assistive devices, or extracorporeal membrane oxygenation (ECMO); and patients who had undergone aortic dissection or thoracotomy surgeries were excluded from the study. Of the 554 patients who underwent cardiac surgery, 243 received third-generation antibiotics (ceftizoxime) and 311 received first-generation antibiotics (cefazolin). According to the exclusion criterion, 27 patients in the third-generation group and 45 patients in the first-generation group were excluded. The final study included 216 patients in the third-generation group and 266 in the first-generation group. Following propensity score matching (PSM), a total of 194 patients were categorized into two groups (Fig. 1). The cephalosporin-based prophylactic antibiotics used were cefazolin (first-generation) and ceftizoxime (third-generation).

### Antibiotic regimen and surgical preparation

A single cephalosporin antibiotic was administered. At least 1 g was administered intravenously over 15 to 20 min, within one hour before the skin incision was made at the sternum, and additional doses were administered at eight-hour intervals for three days postoperatively. Because the timing of prophylactic antibiotic administration is important in preventing SSIs, we defined the antibiotic drug regimen as optimal prophylaxis if the antibiotic was administered within one hour before the first surgical incision. All preoperative procedures were conducted in the same way. The patients were given a chlorhexidine shower before surgery to reduce bacterial proliferation and prevent infection. In the operating room, on the day of surgery, all operative sites were painted with 3 M DuraPrep surgical solution (0.7% iodine-povacrylex, 3 M Health) again. All operations were performed by two experienced cardiac surgeons. The surgical procedures have not been changed to date.

### Surgical site infections

The sternal incision sites were evaluated daily by cardiac surgeons and four times a week by an infection management nurse. The diagnosis of identified SSIs was based on positive cultures, dehiscence of the sternotomy, high fever, local pain, redness, purulent drainage, and sternal instability. The duration of the SSI assessments was within 30 days from the beginning of the follow-up period to the end of follow-up based on the SSI guidelines from the CDC [11]. Nosocomial SSIs were defined according to the CDC criteria and mediastinitis was defined according to the Society of Thoracic Surgeons (STS) criteria [12, 13]. Wound cultures were obtained and clinically processed in the microbiology laboratory according to standard procedures.

### Costs

The total hospital charges included all medical costs covered by health insurance, self-pay, optional care, and other medical costs. To investigate the costs related to SSIs, the pre-cardiac surgery examination costs, imaging costs, nursing costs, surgical costs, material costs, and admission fees were excluded from the total medical cost. All admission fees were reimbursed based on the admission fee for a six-person room. The hospital charges included post-cardiac surgery medication and treatment costs, examination fees, radiology fees, and treatment material costs for the management of early postoperative complications that occurred during hospitalization.

The daily weighted average costs for prophylactic antibiotics were 5.16 US dollars (USD)–5.20 US dollars (USD) for third-generation (ceftizoxime) and 1.08 USD–1.24 USD for first-generation (cefazolin) antibiotics. The exchange rate was based on the rate for November 20, 2019 (1 USD = 1175 Korean won {KRW}).

### Statistical analysis

The continuous variables and categorical variables were analyzed by t-tests and chi-squared tests. PSM was used to control selection bias in group selection. This matching method is designed to compare the individual characteristics of two groups based on propensity scores and conditional probabilities. The first-generation and third-generation groups were matched at a 1:1 ratio using the Greedy matching method [14]. Greedy matching is a method of setting a range of constant propensity scores around a treatment group using a caliper and selecting the closest objects in the control group corresponding to this range. The propensity scores used to estimate the probability of SSI incidence variables were calculated using logistic regression models. The covariates considered in calculating the propensity scores included age, sex, hypertension, obesity, diabetes, and smoking [15]. Differences in the baseline characteristics were evaluated by standardized differences in the matching variables. Standardized differences of > 10% usually represents a meaningful imbalance in the variables between the groups. The c-statistic and Hosmer–Lemeshow goodness-of-fit statistic were used to assess the propensity score model fit.

All outcome variables were compared between the propensity score-matched first-generation and third-generation groups by t-tests for numerical variables and chi-squared tests for categorical variables to determine the effect of SSI. The crude and adjusted odds ratios with 95% confidence intervals (CI) were calculated to investigate the independent effect of prophylactic antibiotics on SSI parameters using logistic regression. Incremental cost-effectiveness ratios were calculated for significantly different variables between the two groups. All data analyses were performed using the statistical program SAS version 9.4 (SAS Institute Inc., Cary NC, USA).

### Ethics statement

The present study protocol was reviewed and approved by the Institutional Review Board of Inje University Sanggye Paik Hospital (approval No. 2017-05-011-003). Informed consent was obtained from all patients before enrollment. Before the start of the study, the hospital research ethics review committee received the review. Related laws and regulations were followed throughout the research period.

## Results

Among the 554 heart surgery procedures performed between January 2009 and December 2016, the general characteristics of 482 study subjects were compared before PSM and 388 patients after matching. Before matching, gender (*p* = 0.028), obesity (*p* = 0.024), hypertension (*p* = 0.042), and EuroSCORE risk assessment scores (*p* < 0.001) were significantly different between the two groups, but there were no statistically significant differences after PSM indicating that matching was balanced. The Hosmer–Lemeshow model goodness-of-fit test statistics were high (c-statistic = 0.63; 95% CI 0.54–0.88) (Table 1).

**Table 1:** General characteristics of study subjects receiving 3rd and 1st generation prophylactic antibiotics

Variables	3rd generation (n = 216)	1st generation (n = 266)	*p* value	SD (%)	3rd generation (n = 194)	1st generation (n = 194)	*p* value	SD (%)
*Preoperative*								
Gender								
Male	130 (60.2)	186 (69.9)	0.028*	− 44.6	121 (62.3)	129 (66.5)	0.682	− 1.1
Female	86 (39.8)	80 (30.1)			73 (37.7)	65 (33.5)		
Age, years	63.4 ± 11.4	64.2 ± 11.9	0.446	− 15.3	63.4 ± 10.8	63.6 ± 12.8	0.596	− 2.2
< 70 years	128 (59.3)	153 (57.5)	0.281	28.6	119 (61.3)	118 (60.8)	0.841	4.9
≥ 70 years	88 (40.7)	113 (42.5)			75 (38.7)	76 (39.1)		
Obesity								
BMI < 25	158 (73.1)	184 (69.2)	0.024*	41.1	143 (73.7)	144 (74.3)	0.955	− 1.6
BMI ≥ 25	58 (26.9)	82 (30.8)			51 (26.3)	50 (25.7)		
Smoker	82 (37.9)	110 (41.3)	0.086	− 28.4	74 (38.1)	70 (36.1)	0.211	7.6
Hypertension	149 (69.1)	204 (76.7)	0.042*	− 48.6	125 (64.4)	131 (67.5)	0.224	− 8.1
Diabetes mellitus (DM)	61 (28.2)	90 (33.8)	0.112	− 13.4	53 (27.3)	52 (26.8)	0.918	0.7
Hypercholesterolemia	24 (11.1)	34 (12.8)	0.441	− 10.2	20 (10.3)	19 (9.8)	0.868	0.7
Dialysis	11 (5.1)	18 (6.8)	0.404	− 9.8	9 (4.6)	10 (5.1)	0.644	− 0.8
Chronic obstructive pulmonary disease	14 (6.5)	21 (7.9)	0.702	0.8	13 (6.7)	14 (7.2)	0.851	− 0.9
Peripheral vascular disease	13 (6.0)	22 (4.7)	0.343	12.0	9 (4.6)	14 (7.2)	0.186	− 7.2
LV dysfunction								
EF < 30% poor	22 (10.2)	25 (9.4)	0.481	2.9	19 (9.8)	21 (10.8)	0.657	− 3.7
EF 30–50% moderate	56 (25.9)	80 (30.1)			51 (26.3)	59 (30.4)		
EF > 50%	138 (63.9)	161 (60.1)			124 (63.9)	114 (58.8)		
EuroSCORE risk index								
Category 1 (≤ 2)	112 (51.8)	137 (51.5)	< 0.001	− 51.5	101 (51.9)	100 (51.5)	0.388	10.6
Category 2 (3–5)	56 (26.0)	102 (38.4)			51 (26.2)	72 (37.1)		
Category 3 (≥ 6)	48 (22.2)	27 (10.1)			42 (21.9)	22 (11.4)		
*Intraoperative*								
Surgical status								
Elective	185 (85.7)	237 (89.1)	0.110	− 7.6	172 (88.7)	174 (89.7)	0.249	9.3
Urgent	24 (11.1)	19 (7.2)			17 (8.7)	14 (7.2)		
Emergency	7 (3.2)	10 (3.7)			5 (2.6)	6 (3.1)		
Type of surgery								
CABG	113 (52.2)	151 (56.7)	0.382	11.4	105 (54.1)	114 (58.7)	0.676	− 0.8
Valve surgery	98 (45.3)	112 (42.1)			86 (44.3)	77 (39.7)		
Combined CV	5 (2.5)	3 (1.1)			3 (1.6)	3 (1.6)		
Internal thoracic artery								
Harvested No	84 (38.9)	111 (41.7)	0.187	− 10.2	73 (37.6)	76 (39.2)	0.280	− 9.8
Harvested left only	106 (49.1)	111 (41.7)			98 (50.5)	87 (44.9)		
Harvested both	26 (12.0)	44 (16.6)			23 (11.9)	31 (15.9)		
Duration of operation								
< 4 h	51 (23.6)	46 (17.3)	0.078	− 12.8	45 (23.2)	46 (23.7)	0.775	0.9
≥ 4 h	165 (76.4)	220 (82.7)			149 (76.8)	148 (76.2)		

Values are presented as the number (%) and mean ± standard deviation. SD = standardized differences as a percentage

*BMI* body mass index, *LV* left ventricle, *EF* ejection fraction, *EuroSCORE* European System for Cardiac Operative Risk Evaluation, *CABG* coronary artery bypass graft, *CV* combined coronary and valve operation

In a comparison of sternal wound infection rates between the two groups, the incidence of superficial SSIs was 9.8% in the first-generation group and 10.3% in the third-generation group (*p* = 0.86). However, in deep SSIs rates in the first-generation antibiotic group (5.7%) was significantly lower than that in the third-generation antibiotic group (16.5%) (*p* < 0.001). In multiple analysis, after adjusting for the variables of sex, age, diabetes, obesity, smoking, emergency, internal thoracic artery(ITA) use, ventilator, year and ICU stay, the incidence of deep SSIs was significantly lower in the first-generation than in the third-generation group (adjusted OR = 1.25, 95% CI 1.07–1.91) (Table 2).

**Table 2:** Clinical outcomes of patients receiving 1st generation prophylactic antibiotics compared to 3rd generation

	3rd generation (n = 194)	1st generation (n = 194)	Crude OR (95% CI)	Adjusted OR (95% CI)
All surgical site infections	52 (26.8)	30 (15.4)	1.19 (1.01–1.71)	1.10 (1.01–1.62)*
Superficial surgical site infection	20 (10.3)	19 (9.8)	0.91 (0.71–1.30)	0.87 (0.62–1.11)
Deep SSI/mediastinitis	32 (16.5)	11 (5.7)	1.36 (1.11–2.08)	1.25 (1.07–1.91)**

Values are presented as the number (%). *p < 0.05, **p < 0.001

Adjustment variables: age, sex, DM, obesity, smoking, emergency, internal thoracic artery (ITA) harvested, ventilator time, duration of ICU stay, and enrollment period

Gram positive bacteria were highly detected and the strain pattern in the both group. Pathogens isolated from the SSIs resulted in that a common infection with β-lactam-resistant gram-positive cocci (e.g., methicillin-resistant *S aureus* and methicillin-resistant *Enterococci*) were significantly less frequent in patients who received first-generation antibiotics (11 of 194 patients (5.6%) than those who received third-generation antibiotics (24 of 194 patients (12.4%), *p* < 0.01). Also, methicillin-susceptible *S. aureus* and coagulase-negative *Staphylococci* were significantly less frequent in the first-generation group (9 of 194 patients (4.4%) than in the third-generation group (25 of 194 patients (7.7%), *p* = 0.028) (see Additional file 1).

The preoperative hospitalization duration and ventilator use time were similar in the two groups at 8.4 $\pm$ 8.6 days for the third-generation group and 7.8 $\pm$ 7.6 days for the first-generation group (*p* = 0.262), and 1.2 ± 2.2 days for the third-generation and 1.2 ± 2.1 days for the first-generation (*p* = 0.679), respectively. However, a significant difference was found in the intensive care unit (ICU) stay duration, with 4.1 ± 3.8 days for the third-generation group and 2.9 ± 2.7 days for the first-generation group (*p* = 0.008). The total hospitalization duration was increased significantly in the third-generation group to 29.8 ± 18.7 days compared to 25.5 ± 20.1 days in the first-generation group (*p* = 0.025) (Table 3).

**Table 3:** Comparison of ICU stay and hospitalization duration in the 1st and 3rd generation groups

Duration	3rd generation (n = 194)		1st generation (n = 194)		*p* value
Hospitalization duration (d)	29.8 ± 18.7		25.5 ± 20.1		0.025
Preoperative hospitalization (d)	8.4 $\pm$ 8.6		7.8 $\pm$ 7.6		0.262
Ventilation use time (d)	1.2 ± 2.2		1.2 ± 2.1		0.679
ICU stay (d)	4.1 ± 3.8		2.9 ± 2.7		0.008
SSI patients ICU stay (d)	n = 52	8.9 ± 4.8	n = 30	7.1 ± 4.7	0.027
Non-infected patients ICU stay (d)	n = 142	2.6 ± 1.2	n = 164	2.3 ± 2.0	0.088

Values are presented as the mean ± standard deviation. *p* values were calculated using the Student’s t-test

*ICU* intensive care unit, *d* day

Compared to medical costs in the two groups, the total cost of daily medical expenses *(p* < 0.001) and the total hospitalization expenses (*p* < 0.001) were increased significantly in the third-generation group. The medical costs for non-infected patients were not statistically different (*p* = 0.092) between two groups but statistical differences for SSIs infected patients were observed in medical costs between the first and third-generation antibiotic groups (*p* < 0.05). The results showed that medical costs were reduced in the first-generation group, at 5894 USD (Table 4).

**Table 4:** Comparison of medical-cost expenditures (USD) in the 1st and 3rd generation groups

Cost	3rd generation (95% CI)		1st generation (95% CI)		*p* value
Antibacterial drug costs	225 ± 92 (210–238)		180 ± 13 (164–192)		< 0.001
Total medical costs/day	1408 ± 760 (1382–1430)		959 ± 738 (944–981)		< 0.001
Total medical costs/admission	26,488 ± 18,402 (26,408–26,524)		20,594 ± 17,206 (20,539–20,667)		< 0.001
SSI patients total medical costs	n = 52	46,154 ± 34,821 (45,984–46,339)	n = 30	27,191 ± 27,190 (26,936–27,311)	0.022
Non-infected patients total medical costs	n = 142	21,251 ± 10,594 (21,187–21,311)	n = 164	18,443 ± 11,701 (18,322–18,512)	0.092

Values are presented as mean ± standard deviation (95% confidence interval). *p* values were calculated using the Student’s t-test

*ICU* intensive care unit, *d* day

## Discussion

The results of this study showed a significantly higher incidence of deep-SSIs and all-SSIs in the third-generation group. The first-generation antibiotic showed excellent antimicrobial effects on β-lactam-resistant gram-positive bacteria and remained stable for long-term at infection rates. In the comparison of hospitalization between the two groups, the preoperative hospitalization duration, mean operation time, and ventilator time were similar in both groups, but the hospitalization duration after surgery was significantly shorter in the first-generation antibiotic group.

This study was conducted to identify the use of prophylactic antibiotics and the source of infections and provide basic data for establishing antibiotic use guidelines. In a previous study, no differences were found in SSI rates after cardiac surgery between the third-generation and first-generation antibiotic groups, although a differences were found between different antibiotic dosage and usage [16, 17]. However, in this study, while no differences in superficial SSI rates were observed between the third- and first-generation groups, significantly lower SSI and deep SSI/mediastinitis rates were found in the first-generation group. Superficial SSIs may be caused by impaired cutaneous circulation, whereas deep SSIs may reflect the relationship between tissue perfusion and infection, including muscle, bone, and the mediastinum in the surgical site and are less frequent than superficial SSIs but have a shorter duration to diagnosis and higher mortality and morbidity. Deep SSIs are one of the most destructive cardiac surgery complications in patients and are different than superficial SSIs. Because the potential infection associations are substantially different, different treatment methods and strategies should be established. Therefore, the high incidence of deep SSIs in the third-generation group was confounded by complex complications and surgical treatment, which lead to longer ICU stays and re-admission rates and doubles the risk of mortality [18]. The effect of SSIs is influenced by antibiotic resistance and the number of infections [19]. Gram-positive bacteria and gram-negative bacteria were cultured from the SSIs of 67% and 23% of the patients in the third-generation group and from 62 and 24% of the patients in the first-generation group, respectively. *S aureus* and coagulase-negative S*taphylococci*, known to be important pathogens responsible for SSIs in heart surgery, are frequently resistant to β-lactam antibiotics [20, 21]. We found that the patients who received third-generation antibiotics for prophylaxis became significantly more colonized with methicillin-resistant coagulase-negative bacteria and *S aureus* than the first-generation group. We observed a trend toward more SSIs in the patients who received third-generation antibiotic prophylaxis. Thus, SSIs caused by methicillin-resistant gram-positive cocci were more common among patients who received third-generation antibiotics.

Considering that the antimicrobial characteristics of the two antibiotics differ, it is appropriate to use first-generation antibiotics because they have excellent antimicrobial activity against gram-positive bacteria and maintain a narrow range of antimicrobial activity. In addition, first-generation antibiotics are more effective in reducing medical costs and increasing safety because they have been used for a long time and are inexpensive. Bratzler et al. [18, 22] warned that the use of prophylactic antibiotics that are incompatible with guidelines is not only less effective in reducing SSIs, but the use of antibiotics over excessively broad antimicrobial ranges may increase the tolerance of other organisms. Barie et al. [23] reported that the choice of the appropriate prophylactic antibiotic is important to cover the range of surgical wound infection organisms and the use of inappropriate prophylactic antibiotics is not effective in reducing surgical wound infection rates. According to Bratzler et al. [18] prophylactic antibiotic selection recommends the use of narrow antibiotic ranges and older-used antibiotics due to factors such as cost, half-life, safety, and antibiotic resistance. Therefore, newer and broader range antibiotics should be avoided as they may increase tolerance. This study did not show any clear advantage of newer and broader range third-generation antibiotics in reducing SSI rates and methicillin-resistant infections in cardiac surgery. In addition, the preoperative conditions, surgical procedures and technique, and antibiotic administration were similar in both groups but differed significantly in their effectiveness to prevent infection. Considering the stability, resistance, and efficacy of the antibiotics, first-generation antibiotics are suitable prophylactic drugs for heart surgery.

The duration of hospital stays in the first-generation group was significantly shorter than in the third-generation group. The preoperative hospital length of stay, operating time, and duration of ventilator use did not differ between the two groups. However, in the first-generation group, the ICU stay and hospitalization duration were both significantly shorter than in the third-generation group. In addition, in a comparison of the length of ICU stay between the SSI groups (n = 82) and the non-infected group (n = 306), the mean duration of ventilator use was 2.5 ± 3.7 days versus 0.99 ± 1.4 days (*p* < 0.001) and the mean ICU stay duration was 7.3 ± 4.8 days versus 2.4 ± 1.4 days (*p* < 0.001), respectively, significantly higher in the infected groups. This result may reflect the increased susceptibility to SSIs with the long-term use of ventilators and increased ICU stay duration, leading to increased treatment due to infection. Lola et al. [24] reported that patients using ventilators for more than 48 h in the ICU had five-fold higher SSI rates and were eight-fold more likely to be readmitted to the ICU due to complications. Therefore, the significant difference in the hospitalization duration between the two groups suggests that long-term ventilator use and ICU stay duration were independent risk factors for SSIs.

Prophylactic antibiotic prices vary slightly from manufacturer to manufacturer, but the first-generation is the oldest drug in the classification of cephalosporins and has the lowest cost. The costs of prophylactic antibiotics may be reflected in the overall cost of patient care and treatment. The total medical care expenditure was about 5800 USD higher in the third-generation group, excluding pre-surgery examination fees, cardiac surgery costs, and material costs for the treatment. In particular, while no difference was observed in total medical expenditures between patients in the non-infected group, a significant difference was found in the patients with SSIs. Third-generation antibiotic prophylaxis affected the length of hospitalization and increased the cost of medical care. This was reflected in increases in the SSI rate, hospitalization duration, and medical expenditures for additional treatments [25]. In addition, if the indirect costs that were not evaluated in this study, were added, SSI could result in significant economic losses.

This study had several limitations as a prophylactic antibiotic study. First, the SSI rate was higher than that of a previous study [17]. The patients were followed up within 30 days of surgery, and SSIs were judged according to the findings of the clinical physician, rather than the infection specialist physician. As such, the clinical physician might have overestimated the incidence of wound infections. Second, while all patients underwent the same surgical procedure, the enrollment period was eight years. Due to the long-term study of eight years, it was analyzed to see if there is a confounding factor by year. The period from 2014 to 2016 taken as a reference year, had the lowest number of infections, so it was analyzed over two years (see Additional file 2). Third, the numbers of enrolled patients with long-term SSIs were insufficient. Further studies are needed to identify additional interrelated risk factors, including variables that can affect SSIs. The prophylactic antibiotic treatment duration and incidence of SSIs need to be established through randomized clinical trials.

## Conclusions

The results of this study showed that the use of third-generation prophylactic antibiotics increased the surgical site infection rate and the length of hospital stay compared to the use of first-generation antibiotics. In addition, the microbial cultures showed that the numbers of gram-positive bacteria and antibiotic resistant organisms at the surgical site were high. It is, therefore, important to select suitable prophylactic antibiotics. The selection of first-generation prophylactic antibiotics, with their long-term safety and low cost, was effective in reducing the rate of surgical site infections and decreasing hospitalization and medical expenditures.

## Supplementary Information

**Additional file 1: Table S1.** Microorganisms isolated according to surgical site infections and prophylactic antibiotics.

**Additional file 2: Table S2.** Clinical outcomes of surgical site infections in two-year intervals.

## Data Availability

The datasets of the current study are available from the corresponding author upon reasonable request.

## Abbreviations

CABG: Coronary artery bypass graft
CPB: Cardiopulmonary Bypass
CI: Confidence interval
ECMO: Extracorporeal Membrane Oxygenation
EuroSCORE: European System for Cardiac Operative Risk Evaluation
HIRA: Health Insurance Review and Assessment Service
ITA: Internal thoracic artery
OR: Odds ratio
PSM: Propensity Score Matching; SSI: Surgical Site infection
