# Supplementary material for: The effect of first- and third-generation prophylactic antibiotics on hospitalization and medical expenditures for cardiac surgery
Source: J Cardiothorac Surg. 2022 Feb 5;17:15. doi: 10.1186/s13019-022-01763-4 (PMC8817574; doi:10.1186/s13019-022-01763-4)
Supplement: Supplementary file 1 — Additional file 1: Table S1. Microorganisms isolated according to surgical site infections and prophylactic antibiotics. [file 13019_2022_1763_MOESM1_ESM.docx]

**Table A1.** Microorganisms isolated according to surgical site infections and prophylactic antibiotics

|  | **3^rd^ generation** | | | | | | **1^st^ generation** | | | |
| --- | --- | --- | --- | --- | --- | --- | --- | --- | --- | --- |
|  | **Superficial SSIs** | | **Deep SSIs** | | **Mediastinitis** | **Total** | **Superficial SSIs** | **Deep SSIs** | **Mediastinitis** | **Total** |
|  | n=20 | | n=24 | | n=8 | n=52 | n=19 | n=6 | n=5 | n=30 |
| **Coagulase-negative *Staphylococci*** | | | | |  |  |  |  |  |  |
| All | | 8 (15.4) | | 9 (17.3) | 5 (9.6) | 22 (42.3) | 8 (26.7) | 3 (10.0) | 4 (13.3) | 15 (50.0) |
| Methicillin-resistant | | 4 (7.7) | | 4 (7.7) | 4 (7.7) | 12 (21.1) | 4 (13.3) | 1 (3.3) | 2 (6.7) | 7 (23.3) |
| Methicillin-susceptible | | 4 (7.7) | | 5 (9.6) | 2 (3.8) | 10 (19.2) | 4 (13.3) | 2 (6.7) | 2 (6.7) | 8 (26.7) |
| ***Staphylococcus aureus*** | | | | |  |  |  |  |  |  |
| All | | 6 (11.5) | | 8 (15.4) | 3 (5.8) | 17 (32.7) | 3 (10.0) | 1 (3.3) | 1 (3.3) | 5 (16.7) |
| Methicillin-resistant | | 3 (5.8) | | 5 (9.6) | 2 (3.8) | 10 (19.2) | 1 (3.3) | 1 (3.3) | 1 (3.3) | 3 (10..0) |
| Methicillin-susceptible | | 2 (3.8) | | 2 (3.8) | 1 (1.9) | 5 (9.6) | 1 (3.3) |  |  | 1 (3.3) |
| *Enterococci* | | 1 (1.9) | | 1 (1.9) |  | 2 (3.8) | 1 (3.3) |  |  | 1 (3.3) |
| **Gram-negative bacilli** | | | |  |  |  |  |  |  |  |
| All | | 4 (7.7) | | 7 (13.5) |  | 11 (21.2) | 6 (20.0) | 2 (6.7) |  | 8 (26.7) |
| *Klebsiella* species | | 2 (5.8) | | 3 (5.8) |  | 5 (9.6) | 3 (10.0) | 1 (3.3) |  | 4 (13.3) |
| *Enterobacter* species | |  | | 1 (1.9) |  | 1 (1.9) |  |  |  |  |
| *Acinetobacter* species | | 1 (1.9) | | 2 (3.8) |  | 3 (5.8) | 2 (6.7) | 1 (3.3) |  | 3 (10.0) |
| *Pseudomonas* species | | 1 (1.9) | | 1 (1.9) |  | 2 (3.8) | 1 (3.3) |  |  | 1 (3.3) |
| **Culture negative** | | 2 (3.8) | |  |  | 2 (3.8) | 2 (6.7) |  |  | 2 (6.7) |
| Values are presented as the number (%). | | | | | | | | | | |
