# Supplementary material for: The effect of first- and third-generation prophylactic antibiotics on hospitalization and medical expenditures for cardiac surgery
Source: J Cardiothorac Surg. 2022 Feb 5;17:15. doi: 10.1186/s13019-022-01763-4 (PMC8817574; doi:10.1186/s13019-022-01763-4)
Supplement: Supplementary file 2 — Additional file 2: Table S2. Clinical outcomes of surgical site infections in two-year intervals. [file 13019_2022_1763_MOESM2_ESM.docx]

**Table A2.** Clinical outcomes of surgical site infections in two-year intervals

|  | All SSIs (n=82) | Superficial SSIs (n=39) | Deep SSIs/mediastinitis (n=43) |
| --- | --- | --- | --- |
|  | OR (95% CI) | OR (95% CI) | OR(95% CI) |
| Enrollment period |  |  |  |
| 2014 – 2016 | Ref | Ref | Ref |
| 2012 – 2014 | 0.84 (0.56-1.41) | 0.79(0.42-1.22) | 1.02(0.67-1.46) |
| 2010 – 2012 | **1.12 (1.00-2.03) *** | 0.87(0.55-1.56) | **1.21(1.07-2.23)*** |
| 2009 – 2010 | **1.31(1.11-2.48)**** | 1.02(0.91-1.45) | **1.56(1.22-2.67)**** |
| *p < 0.05, **p < 0.001  Adjustment variables: age, sex, DM, obesity, and smoking  -Since the infection rate was lowest from 2014 to 2016, which is the reference year, it was analyzed over two years. | | | |
